# HIV-2 Integrase Polymorphisms and Longitudinal Genotypic Analysis of HIV-2 Infected Patients Failing a Raltegravir-Containing Regimen

**DOI:** 10.1371/journal.pone.0092747

**Published:** 2014-03-28

**Authors:** Joana Cavaco-Silva, Ana Abecasis, Ana Cláudia Miranda, José Poças, Jorge Narciso, Maria João Águas, Fernando Maltez, Isabel Almeida, Isabel Germano, António Diniz, Maria de Fátima Gonçalves, Perpétua Gomes, Celso Cunha, Ricardo Jorge Camacho, on behalf of the Portuguese HIV-2 Resistance Study Group

**Affiliations:** 1 Centro de Malária e Outras Doenças Tropicais, Unidade de Microbiologia Médica, Instituto de Higiene e Medicina Tropical, Universidade Nova de Lisboa, Lisboa, Portugal; 2 Centro de Malária e Outras Doenças Tropicais, Unidade de Saúde Pública Internacional e Bioestatística, Instituto de Higiene e Medicina Tropical, Universidade Nova de Lisboa, Lisboa, Portugal; 3 Serviço de Doenças Infecciosas, Centro Hospitalar de Lisboa Ocidental, Lisboa, Portugal; 4 Hospital de São Bernardo, Centro Hospitalar de Setúbal, Setúbal, Portugal; 5 Hospital dos SAMS, Lisboa, Portugal; 6 Hospital Garcia de Orta, Almada, Portugal; 7 Hospital Curry Cabral, Centro Hospitalar de Lisboa Central, Lisboa, Portugal; 8 Hospital de Santo António, Centro Hospitalar do Porto, Porto, Portugal; 9 Hospital de São José, Centro Hospitalar de Lisboa Central, Lisboa, Portugal; 10 Hospital Pulido Valente, Centro Hospitalar de Lisboa Norte, Lisboa, Portugal; 11 Laboratório de Microbiologia Clínica e Biologia Molecular, Serviço de Patologia Clínica (SPC), Centro Hospitalar de Lisboa Ocidental, Lisboa, Portugal; 12 Centro de Malária e Outras Doenças Tropicais, Instituto de Higiene e Medicina Tropical, Universidade Nova de Lisboa, Lisboa, Portugal; 13 Centro de Investigação Interdisciplinar Egas Moniz (CiiEM), Instituto Superior de Ciências da Saúde Sul, Caparica, Portugal; 14 Rega Institute for Medical Research, Katholieke Universiteit Leuven (KU Leuven), Leuven, Belgium; University of Pittsburgh, United States of America

## Abstract

To characterize the HIV-2 integrase gene polymorphisms and the pathways to resistance of HIV-2 patients failing a raltegravir-containing regimen, we studied 63 integrase strand transfer inhibitors (INSTI)-naïve patients, and 10 heavily pretreated patients exhibiting virological failure while receiving a salvage raltegravir-containing regimen. All patients were infected by HIV-2 group A. 61.4% of the integrase residues were conserved, including the catalytic motif residues. No INSTI-major resistance mutations were detected in the virus population from naïve patients, but two amino acids that are secondary resistance mutations to INSTIs in HIV-1 were observed. The 10 raltegravir-experienced patients exhibited resistance mutations via three main genetic pathways: N155H, Q148R, and eventually E92Q - T97A. The 155 pathway was preferentially used (7/10 patients). Other mutations associated to raltegravir resistance in HIV-1 were also observed in our HIV-2 population (V151I and D232N), along with several novel mutations previously unreported. Data retrieved from this study should help build a more robust HIV-2-specific algorithm for the genotypic interpretation of raltegravir resistance, and contribute to improve the clinical monitoring of HIV-2-infected patients.

## Introduction

The Human Immunodeficiency Virus Type 2 (HIV-2) infects 1–2 million people worldwide, most of which in West Africa, where it is endemic [1]. In Europe, Portugal is the country with the highest frequency of HIV-2 infection, accounting for 543 (3.1%) of all AIDS cases [2]. Despite a slow progression of disease in most infected people, 20–25% of HIV-2 patients progress to AIDS if untreated [3], [4].

The therapeutic options for HIV-2 patients are still limited. The virus is naturally resistant to non-nucleoside reverse transcriptase inhibitors (NNRTIs) [5], [6] and to the fusion inhibitor enfuvirtide (T-20) [6]–[8]. Compared to HIV-1, HIV-2 has reduced sensitivity to some protease inhibitors (PIs) [9], [10], a lower genetic barrier towards resistance for most drugs [11]–[13], along with a faster acquisition of resistance for some of them [12].

The recent pharmacological class of Integrase Strand Transfer Inhibitors (INSTIs) represents a promising therapeutic option for the treatment of HIV-2 infection. Integrase (IN) is a multi-domain protein consisting of the N-terminal domain (NTD, HIV residues 1–49), catalytic core domain (CCD, residues 50–212), and C-terminal domain (CTD, residues 213–288). The NTD contains a conserved HHCC Zn-coordination motif, and Zn-binding contributes to IN multimerization and catalytic function [14], [15]. The CCD contains an invariant triad of acidic residues (Asp-64, Asp-116, Glu-152 on HIV-1) that forms the enzyme active site [16]–[18]. The CCD also contributes to IN multimerization [19] and engages viral [20]–[22] and chromosomal [23], [24] DNAs during integration. The CTD, which is the least conserved of the domains among retroviruses [25], also contributes to specific [26] and non-specific [27]–[29] DNA interactions, as well as multimerization [30].

Despite a 40% difference in amino acid sequence between HIV-1 and HIV-2 integrases, phenotypic assays carried out with reference strains or clinical isolates have shown that all approved inhibitors [i.e. raltegravir (RAL), elvitegravir (EVG) and dolutegravir (DTG)] are effective against HIV-2. *In vitro* studies have shown that the HIV-1 and HIV-2 wild-type integrases have a similar phenotypic susceptibility to INSTIs [31]–[34], probably due to the 100% conservation of the catalytic triad DDE and the HHCC and RKK motifs between the two viruses [33], [35], [36,36], and that INSTIs exert a potent inhibitory effect on HIV-2 replication [33], [37]. *In vivo* data has also revealed promising results: raltegravir, the first INSTI to be licensed for clinical use, has been used on some HIV-2 infected patients as part of their salvage regimens with, at least, a good short term response concerning suppression of plasma viral load and CD4 cell recovery [34], [38]–[40]. However, as for other antiviral drugs, resistance to RAL emerges rapidly both *in vitro* and *in vivo*, greatly reducing the susceptibility of the virus to the inhibitor. The specific amino acid changes that are known to confer RAL resistance in HIV-1 have also been shown to develop in RAL-treated HIV-2 patients: N155H, Q148K/R and, to a lesser extent, Y143C [39], [41]–[46]. Overall, three major resistance pathways have been identified and shown to elicit high-level raltegravir and elvitegravir resistance in HIV-2: i) N155H/E92Q, ii) Q148R/G140S, and iii) Y143C/E92Q or Y143C/T97A [42], [47], [48]. On the other hand, the N155H-Y143C and N155H-Q148R replacements seem to define mutually exclusive pathways to RAL resistance in HIV-2 [32], [42]. RAL and EVG show extensive cross-resistance in HIV-2, as in HIV-1 [43], [46], [49], [50], but DTG retains activity against some RAL and EVG-resistant HIV-1 strains, both in *vitro* and *in vivo* [51]–[53]. Information on its efficiency against HIV-2 strains bearing integrase inhibitor resistance mutations is still limited, but phenotypic assays carried out with HIV-2 clinical isolates from patients treated with RAL showed that mutations T97A/Y143C, G140S/Q148R or G140T/Q148R/N155H conferred moderate resistance to DTG (7–18-fold increase of the EC_50_) [37]. As in HIV-1 the association T97A/Y143C does not confer resistance to DTG, extreme caution must be taken when extrapolating HIV-1 knowledge to HIV-2. Corroborating this is the fact that, at this time, it is still unknown whether the combination H51Y/R263K, that confers some level of resistance to DTG in HIV-1 [54], [55], could be relevant as a mutational pathway leading to HIV-2 resistance.

Overall, HIV-2 resistance pathways to INSTIs are still poorly characterized. So the aims of this study were i) to investigate the natural polymorphism of the HIV-2 integrase gene, and ii) to further characterize the genotypic resistance profiles of HIV-2 patients failing RAL-containing regimens.

## Materials and Methods

### Study design and sequences

This study assessed 73 HIV-2 group A infected patients followed at different hospitals mainly in the central and southern areas of Portugal. The HIV-2 group was determined using the Rega Subtyping Tool v2.0. 63 patients were RAL-naïve, and constituted the control group. 23/63 had been previously exposed to antiretroviral (ARV) therapy: 21 had received nucleoside/nucleotide reverse transcriptase inhibitors (NRTIs) and protease inhibitors (PIs), one had received NRTIs and non-nucleoside reverse transcriptase inhibitors (NNRTIs), and one had received NRTIs, NNRTIs and PIs. The remaining 40/63 patients had never been exposed to antiretroviral drugs at the time the sample was obtained. Ten patients were heavily pretreated and were on a salvage RAL-containing regimen. All the longitudinal genotypic resistance data available for these patients, pre- and during RAL exposure, was considered for analysis, in a total of 20 sequences. For statistical purposes, only one genotype, corresponding to the most recent isolate of each patient, was considered.

Mutations were defined as differences at the amino acid level from the wild-type reference sequence (HIV2_ROD_M15390), including those that were present as part of a nucleotide mixture. Despite not being a primary isolate, HIV-2_ROD can be considered a reference control isolate for this study because its susceptibility to INSTIs has been previously demonstrated [33].

The sequences obtained from RAL-naïve patients were used to uncover natural polymorphisms, and to determine whether the critical amino acid substitutions associated with resistance to INSTIs in HIV-1 are naturally present in HIV-2. An amino acid position was considered polymorphic when at least two different amino acids were found [56]. Amino acids not mutated were defined as conserved. Amino acid variability at a given position was established by calculating the percentage of sequences harboring an amino acid different from the HIV2_ROD_M15390 reference sequence. The most polymorphic positions were considered to be those where more than 30% of sequences in the control group were variable, according to the criterion previously defined by others [56].

Mutations were considered as being selected by therapy if absent from RAL-naïve sequences and present in ≥1 sequences from RAL-treated individuals. All the amino acid variants occurring at non-polymorphic HIV-2 integrase positions, i.e., occurring only in RAL-treated individuals, were analyzed. We also investigated the occurrence at such positions of major and compensatory drug resistance mutations already associated with *in vivo* or *in vitro* resistance to INSTIs in HIV-1 [according to Lataillade M et al. 2007 [35] and to the 2013 Update of the Drug Resistance Mutations in HIV-1 [57]] or in HIV-2 [according to the Algorithm for the Interpretation of Genotypic HIV-2 Resistance Data (http://regaweb.med.kuleuven.be/sites/default/files/algorithms/Rega_HIV2_Rules_v8.0.2.pdf) [58]].

### HIV-2 integrase sequencing

The integrase coding region of the *pol* gene was amplified and sequenced from plasma samples of the HIV-2 infected patients, in a total of 293 amino acids analyzed. Viral RNA was extracted from 1 ml of plasma according to *Biomérieux*'s *easyMAG* automatic extraction procedure, and used to amplify and sequence the last domain of the *pol* gene, comprising the integrase. To do so, the protocol described at Bercoff DP *et al.* [36] was used, but some adjustments were made. 10–15 µl of RNA were initially subjected to 5 min at 70°C followed by 5 min on ice to avoid the generation of possible intramolecular base-pairings in single-stranded RNAs. RNA was then retrotranscribed using *Super SuperScript III One-Step RT-PCR System with Platinum Taq DNA Polymerase* (*Invitrogen Life Technologies*) in a 50 µl mix containing 10 µM of each forward primer JR25 and reverse primer JR47 (**Table 1**). Cycling conditions consisted of reverse transcription at 45°C for 1 h, denaturation at 94°C for 2 min followed by 40 amplification cycles of 94°C for 30 sec, 53°C for 45 sec and 72°C for 1 min 45 sec, and a final elongation step at 72°C for 3 min. A nested PCR was then performed with 2 µl of the previously amplified cDNA, using AmpliTaq Gold DNA Polymerase (*Applied Biosystems*) with 10 µM of each forward primer H2Mp9 and reverse primer JR46 (**Table 1**). Nested PCR conditions consisted of denaturation at 95°C for 10 min, 40 amplification cycles of 94°C for 30 sec, 50°C for 45 sec and 72°C for 1 min 30 sec, and a final elongation step at 72°C for 10 min. Amplification products were checked on a 2% agarose gel with ethidium bromide and 8 µL of GeneRuler DNA Ladder Mix (*Fermentas*) for reference, and were subsequently purified through the YM-100 Microconcentrators Method according to the *ViroSeq HIV-1 Genotyping System v2.0* Protocol (*Abbott*). Dilution adjustments of the PCR products were made when necessary. The sequencing reaction was then performed using the *Big Dye Terminator v3.1 Cycle Sequencing Kit (Applied Biosystems)* and primers H2Mp9, JR44, JR45, AV33 (forward), JR46 and JR48 (reverse) (**Table 1**). Cycling conditions consisted of 30 amplification cycles of 10 sec at 96°C, 5 sec at 50°C and 4 min at 60°C. Products were purified and run on an ABI PRISM 3100 Genetic Analyzer (*Applied Biosystems*). Nucleotide sequences were aligned against the HIV-2 ROD reference strain (GenBank accession # M15390), and edited with *SeqScape Software Version 2.5* (*Applied Biosystems*).

**Table 1:** HIV-2 integrase primers given with their positions in HIV-2 ROD indicated in parenthesis. (+, sense; −, antisense).

Primer	Nucleotide sequence	Nucleotides	Reference
JR25	[+2528] 5′-GCA CCT CCA ACT AAT CCT-3′	18	Bercoff DP *et al.* 2010 [36]
JR44	[+3689] 5′-GAG ACC TTC TAC ACA GAT GG-3′	20	Bercoff DP *et al.* 2010 [36]
JR45	[+3971] 5′-TAT GTT GCA TGG GTC CCA GC-3′	20	Bercoff DP *et al.* 2010 [36]
JR46	[−5019] 5′-ATG CCC ATC CCA CCT TAT GGT G-3′	22	Bercoff DP *et al.* 2010 [36]
JR47	[−5041] 5′-ATT ACC CTG CTG CAA CTG CAC C-3′	22	Bercoff DP *et al.* 2010 [36]
JR48	[−4466] 5′-GTT CTA TAC CTA TCC ACC-3′	18	Bercoff DP *et al.* 2010 [36]
AV33	[+4433] 5′-GTG AAG ATG GTA GCA TGG TGG-3′	21	Bercoff DP *et al.* 2010 [36]
H2Mp9	[+2932] 5′-GGA TGA TAT CTT AAT AGC TAG-3′	21	Colson P *et al.* 2004 [56]

### GenBank accession numbers

All the newly determined nucleotide data were deposited in GenBank under the following accession numbers: KF859774 - KF859846, and KF951329-KF951338.

### Statistical analysis

For the determination of which mutations are selected in HIV-2 as a response to RAL pressure, sequences of 63 RAL-naïve and 10 RAL-experienced patients were used, and the prevalence of different amino acids at every codon position was estimated. To do so, the different amino acids occurring at each integrase position were weighted according to the following criterion: when the amino acid was different from that of the reference sequence, it was counted; when the amino acid was the same as the reference sequence, it was not. In case of double and triple populations, only the mutant amino acids were taken into account when counting: each was given the same weight so that the sum of all mutant amino acids at each position would be 1. This was computed by alignment of nucleotide sequences and HIV2_ROD strain with Muscle [59], manual edition with JalView version 2 [60], and writing of the scripts with Python Programming Language (http://www.python.org/) [61]. All integrase positions were compared to the HIV2_ROD_M15390 sequence.

INSTI-naïve patients were further subdivided into a group of ARV-naïve patients and a group of previously ARV (NRTI/PI) - experienced patients, for the purpose of comparing the prevalence of mutated amino acids between both groups. To do so, a χ^2^ test (based on a 2×2 contingency table containing the numbers of isolates from untreated and treated persons, and the number of isolates with and without mutations) was computed in R v.2.15.2, and a p-value<0.05 was considered statistically significant.

Shannon's entropy at each position of INSTI-naïve sequences, a measure of the amount of information contained at that position, was calculated using the Los Alamos Database sequence Entropy website http://www.hiv.lanl.gov/content/sequence/ENTROPY/entropy_one.html for group A strains.

### Ethics Statement

Due to the fact that this is a retrospective study, and that all the procedures were standard of care – e.g., no extra blood collections were performed for study procedures, all the samples being surplus from collections taken for clinical monitoring – the Ethical Committee of Centro Hospitalar de Lisboa Ocidental did not consider to be necessary to obtain written informed consent from patients.

This study received ethical approval by the Ethical Committee of Centro Hospitalar de Lisboa Ocidental (Reference no. 96/CES-2013).

## Results

In the present study, we assessed a total of 83 HIV-2 integrase sequences, which, to our knowledge, represents the most extensive analysis performed to date of the integrase coding region of HIV-2. All the sequences belong to HIV-2 group A. 63 sequences belong to 63 RAL-naïve patients, and 20 sequences belong to 10 heavily pretreated HIV-2 infected patients with virological failure while receiving a salvage raltegravir-containing regimen (**Table 2**).

**Table 2:** HIV-2 group A IN polymorphisms.

		Position ROD	Shannon entropy	RAL-naïve patients (63 sequences)
*30–50%*	**N-terminal Domain HHCC Zinc coordination finger**	L2	0.824	I (19%)
**51–70%**		K4	0.803	N (1%), S (1%), R (24%)
71–90%		Y15	0.571	F (10%)
		*V19*	*1.058*	*I (29%), M (8%)*
		K20	0.665	Q (10%), R (3%)
		S23	1.037	A (8%), C (10%), T (3%)
		**I28**	**0.991**	**M (1%), L (50%)**
		N30	1.279	K (60%), M (2%), L (2%), Q (13%), T (6%)
		L31	0.554	I (5%)
		V32	0.373	I (2%)
		R34	0.780	K (14%)
		S39	0.533	T (92%)
		*A41*	*1.075*	*G (2%), N (2%), P (14%), T (17%)*
		A49	0.266	P (2%)
	**Catalytic Core Domain (with DDE Catalytic Triad)**	I50	0.682	M (5%), T (8%), V (5%)
		(*) H51	0.079	Q (2%)
		N55	0.308	D (10%)
		E57	0.493	A (2%), D (8%)
		L58	0.923	F (2%), I (8%), V (8%)
		T60	0.651	A (1%), I (9%), M (2%), V (3%)
		E69	0.373	D (2%)
		(*) I72	*0.980*	*V (40%)*
		(*) I84	0.266	V (6%)
		E87	0.137	K (2%)
		**S93**	**0.937**	**T (57%)**
		R95	0.576	K (2%)
		Q96	0.349	H (6%), Y (2%)
		L101	0.308	I (2%)
		S106	0.349	G (2%)
		I110	0.079	V (2%)
		T111	0.137	R (2%)
		L113	0.531	V (2%)
		H114	0.231	Q (2%)
		A119	0.536	D (2%), G (2%), P (8%), R (1%)
		T122	0.231	I (2%), M (1%), V (1%)
		Q124	0.450	H (2%)
		(*) E125	0.386	D (2%)
		V129	0.216	A (2%)
		I133	1.104	A (2%), T (3%), V (74%)
		(*) S138	0.582	T (19%)
		V141	0.386	I (2%)
		(*) A153	0.274	S (3%)
		(*) H156	0.388	L (2%), P (2%), R (2%)
		(*) H157	0.310	P (2%)
		(*) S163	0.913	D (21%), G (1%), N (7%)
		R164	0.406	K (10%)
		*E167*	*0.921*	*D (41%)*
		N170	0.464	E (2%), I (2%)
		I172	1.056	M (10%), V (65%)
		E173	0.310	K (2%)
		(*) I175	0.531	L (2%), V (17%)
		L177	0.450	V (2%)
		M178	0.159	I (2%), R (2%)
		I180	0.773	A (6%), T (5%), V (78%)
		H181	0.079	Y (2%)
		C182	0.079	Y (2%)
		R188	0.079	W (2%)
		G189	0.079	E (2%)
		G190	0.079	E (2%)
		**S197**	**0.781**	**A (65%)**
		L200	0.464	F (2%), V (2%)
		(*) I201	0.386	T (2%), V (2%)
		(*)T206	*0.895*	*A (41%), S (2%)*
		E207	0.137	D (3%)
		I210	0.266	V (6%)
		F212	0.271	L (2%)
	**C-terminal Domain**	F213	0.187	F (5%)
		Q214	0.454	H (10%)
		A215	0.788	N (2%), S (10%), T (11%)
		K216	0.079	R (2%)
		D217	0.216	D (2%), K (2%)
		S218	0.476	L (13%)
		K219	0.374	E (2%), N (2%), R (5%)
		*L220*	*0.629*	*F (33%)*
		K221	0.608	Q (19%), R (2%)
		N222	0.419	K (11%)
		R224	0.231	Q (6%)
		F227	0.079	Y (2%)
		R228	0.079	K (2%)
		E229	0.137	K (2%)
		Q233	0.079	H (2%)
		L234	0.080	Q (2%)
		W235	0.079	S (2%)
		E240	0.688	D (2%), K (3%), Q (2%)
		L241	0.159	H (2%)
		*E246*	*0.713*	*D (33%)*
		(*) V249	0.295	I (5%)
		L250	0.556	I (87%), V (1%)
		V251	0.349	A (8%)
		T255	0.744	A (27%)
		D256	0.680	E (11%)
		**I259**	**0.870**	**V (59%)**
		**I260**	**0.949**	**M (1%), V (52%)**
		I267	0.310	V (2%)
		I268	0.216	V (3%)
		*R269*	*0.940*	*K (33%)*
		R274	0.388	K (2%)
		Q275	0.159	K (2%)
		E276	0.344	G (2%)
		**M277**	**1.090**	**L (43%), V (6%)**
		D278	0.159	G (2%)
		S279	0.431	C (2%), N (5%)
		(*) G280	0.680	S (19%)
		**S281**	**0.906**	**P (41%)**
		H282	0.597	N (12%), R (2%)
		L283	0.630	V (8%)
		G285	0.431	D (2%), S (5%)
		*A286*	*0.879*	*T (30%)*
		D289	0.466	N (8%), T (2%)
		E291	0.349	G (2%)
		M292	0.791	V (73%)

Polymorphisms of the HIV-2 group A IN sequences from 63 treatment-naïve patients are reported with respect to the ROD reference sequence. The three different domains of IN are indicated: the N-terminal domain (AA 1–49); the catalytic core domain (AA 50–212) containing the conserved catalytic triad (DDE motif); and the C-terminal domain (AA 213–288). Positions known to confer resistance to INSTIs in HIV-1 or HIV-2 are marked with a star (*). The frequency (percentage) of each of each of the polymorphisms is indicated in brackets. Only positions where variations were detected are reported. Shannon's entropy at each variable position is indicated; an entropy value of 0 corresponds to an amino acid strictly conserved, and higher entropy values indicate more variability.

### Analysis of HIV-2 integrase gene polymorphisms

We analyzed 63 integrase sequences retrieved from RAL-naïve patients. At the time of integrase genotyping, 40 patients were antiretroviral (ART)-naïve, 22 patients were receiving NRTI+PI-based regimens, and one patient was receiving an NRTI+NNRTI-based regimen, despite the widely recognized inefficacy of NNRTIs on HIV-2. We report IN polymorphisms for this group of patients with respect to the reference sequence ROD (**Table 2**).

From a total of 293 integrase codons analyzed, 61.4% (180/293) were non-polymorphic and 38.6% (113/293) were naturally polymorphic. Of the 113 variable positions, 39 (34%) were mutated at least twice (**Table 2**). The catalytic triad DDE, the zinc coordination motif HHCC, and the RKK motif, at positions 64, 116 and 152 (DDE), 12, 16, 40 and 43 (HHCC), and 231, 258 and 264 (RKK), respectively, were completely conserved in our dataset, and the CTD was the least conserved of the three domains, as foreseeable. The most polymorphic positions were located at 23 HIV-2 group A IN positions: 19, 28, 30, 39, 41, 72, 93, 133, 167, 172, 180, 197, 206, 220, 246, 250, 259, 260, 269, 277, 281, 286 and 292 (**Table 2**).

Integrase sequences from RAL-naïve patients were further examined to determine whether the critical amino acid substitutions associated with *in vivo* and *in vitro* INSTIs resistance in HIV-1 [35], [57] occur as baseline polymorphisms in HIV-2. None of the primary mutations at positions 66, 92, 143, 147, 148 and 155 responsible for RAL or EVG resistance in HIV-1 were detected in sequences from HIV-2 RAL-naïve patients. However, two secondary mutations corresponding to resistance-associated substitutions in HIV-1 were observed: 206S and 249I. The most common resistance associated residue was 249I, present in 5% of sequences from the control group, while 206S was present in 2% of these sequences. In this group of patients we also detected polymorphisms at 8 additional positions associated with INSTIs resistance in HIV-1: 125D, 138T, 153S, 156L/P/R, 157P, 163D/G/N, 201T/V and 280S. In these patients, we further observed two amino acid changes that were previously described as secondary mutations in the HIV-2 IN, I84V and S163G/D [44], [45], and two polymorphisms at positions previously associated with HIV-2 INSTIs resistance: H51Q, I175L/V [36], [44], [46].

To evaluate whether specific drug pressure induced by antiviral drugs different from INSTIs, in particular NRTIs, may also select or induce mutations in the HIV-2 IN gene, the different prevalence of IN mutations between the two populations of INSTI-naïve patients (drug naïve and NRTI/PI treated) was investigated. Overall variability within IN was higher in treatment-naïve (34.5%) than in NRTI/PI-experienced patients (23.9%), and this difference reached statistical significance (χ^2^ = 7.43; p = 0.00641). When analyzing each polymorphism individually, we observed that two polymorphisms showed a significant increase in prevalence in HIV-2 NRTI/PI-treated patients compared with ARV naïve patients: V19M (n = 4/23 *vs.* n = 1/40; p = 0.0353) and L58V (n = 4/23 *vs.* n = 1/40; p = 0.0353). Interestingly, and unlike the above-mentioned mutations, other two IN polymorphisms showed a significant decrease in prevalence in isolates from NRTI/PI-treated patients, as compared with drug-naïve patients, thus suggesting a negative association with NRTI/PI treatment: E246D (n = 11/23 *vs.* n = 31/40; p = 0.0162) and M277L (n = 6/23 *vs.* n = 21/40; p = 0.0412).

### Analysis of HIV-2 integrase gene mutations potentially associated with RAL resistance

Ten heavily pretreated HIV-2 infected patients exhibiting incomplete viral suppression or virological rebound while receiving a salvage raltegravir-containing regimen were investigated. Six patients had only one isolate for analysis, and the other four patients had longitudinal genotypic data. In these four patients, RAL was kept despite a detectable viral load, because there were no alternatives to the current regimen. The median time of exposure to RAL was 17 months (range: 8–35 months). When the drug resistance testing was performed, nine patients were receiving different combinations of NRTI+PI, and one patient was receiving only NRTI-based therapy, in addition to RAL. All had previously received several combinations of NRTIs and PIs (**Table 3**).

**Table 3:** Antiretroviral regimens, available genotypes and respective integrase mutations of the ten HIV-2-infected patients on a salvage RAL-containing regimen.

Patient	Therapeutic regimen	Genotype	Plasma HIV-2 RNA (copies/ml)	Exposure time to RAL (months)	HIV-2 integrase mutations		
					Major Mutations	Minor mutations previously reported in the HIV-2 integrase	Minor mutations previously unreported in the HIV-2 integrase (observed in non-polymorphic sites of HIV-2 wild-type, i.e., present only in RAL-treated patients)(b)
**1**	d4T+3TC+IDV/r						
	AZT+TDF+LPV/r						
	ABC+TDF+LPV/r+SQV/r						
	AZT/3TC+TDF+LPV/r+SQV/r						
	TDF/FTC+DRV/r+**RAL**	1	2692	5	Q148R(a)	―	―
**2**	AZT/3TC+TDF						
	TDF/FTC+SQV/r						
	TDF/FTC+**RAL**	1	12989	14	N155H(a)	I84V, E92A, A153G	Q44H
**3**	AZT/3TC+IDV/r						
	AZT/3TC+NFV/r						
	AZT/3TC+IDV/r						
	AZT/3TC+NFV/r						
	AZT/3TC+IDV/r						
	AZT/3TC+LPV/r						
	AZT+TDF+ATV/r						
	3TC+DRV/r+**RAL**	1	9896	8	N155H(a)	I84V, A153G	―
**4**	AZT						
	ddI						
	d4T+3TC+SQV/r						
	ABC+TDF+LPV/r						
	ddI+DRV/r+**RAL**	1	88756	17	N155H(a)	E92Q(a), T97A(a), S163D	Q44H, Q45H, K71R, K127R
**5**	AZT/3TC						
	AZT/3TC+IDV/r						
	ddI+NFV/r						
	TDF+ABC+LPV/r						
	TDF/FTC+DRV/r+**RAL**	1	8313	14	N155H(a)	I84V, E92A, A153G	D232N(a), K236T
**6**	AZT/3TC+IDV/r						
	ABC+ddI+NFV/r						
	d4T+3TC+TDF+LPV/r+APV/r						
	d4T+3TC+TDF+LPV/r+SQV/r						
	d4T+3TC+TDF+ATV/r+SQV/r						
	TDF/FTC+AZT+DRV/r+**RAL**	1	151	5	N155H(a)	I84V, E92Q(a)	―
		2	1107	15	N155H(a)	I84V, E92Q(a)	―
		3	576	22	N155H(a)	I84V, E92Q(a), H157N/S/R	K46R
	TDF/FTC+d4T+DRV/r+**RAL**	4	1074	30		I84V, E92Q(a), H157S	K46R
**7**	AZT/3TC+IDV/r						
	d4T+ddI+IDV/r						
	AZT/3TC+LPV/r						
	AZT+3TC+TPV/r						
	TDF+DRV/r+**RAL**	1	26898	12	N155H(a)	―	L242P
		2	13809	14	N155H(a)	Q91R	K46R, D232N(a)
		3	22692	23	N155H/N(a)	E92E/Q(a)	K46R
		4	16035	35	―	E92Q(a), T97A(a), N160K	K46R
**8**	AZT						
	ddI						
	d4T+3TC+RTV						
	d4T+3TC+IDV/r						
	d4T+3TC+LPV/r						
	TDF/FTC+SQV/r+LPV/r						
	ABC+TDF+TPV/r+**RAL**	1	10079	3	―	―	―
		2	5844	10	N155H(a)	E92G	―
		3	3732	21	N155H(a)	E92G, N160K	K46R, V151I(a)
		4	6715	22	N155H(a)	E92G, N160K	K46R
**9**	AZT/3TC						
	d4T+ddI+IDV/r						
	d4T+ddI+NFV/r						
	d4T+ddI+LPV/r						
	TDF+ABC+fAPV/r						
	TDF/FTC+ABC+ATV/r+SQV/r						
	TDF/FTC+DRV/r+**RAL**	1	ND	12		E92Q(a), T97A(a)	E85K
**10**	AZT/3TC						
	AZT/3TC+IDV/r						
	ddI+NFV/r						
	TDF+ABC+LPV/r						
	TDF/FTC+DRV/r+**RAL**	1	5012	21		―	―
		2	37119	32		E92Q(a), T97A(a)	―

(a) Mutations associated with INIs resistance in HIV-1.

(b) Mutations of unknown impact on RAL resistance.

d4T, stavudine; 3TC, lamivudine; AZT, zidovudine; TDF, tenofovir; ABC, abacavir; FTC, emtricitabine; ddI, didanosine; IDV/r, indinavir boosted with ritonavir; LPV/r, lopinavir boosted with ritonavir; DRV/r, darunavir boosted with ritonavir; SQV/r, saquinavir boosted with ritonavir; NFV/r, nelfinavir boosted with ritonavir; ATV/r, atazanavir boosted with ritonavir; APV/r, amprenavir boosted with ritonavir; TPV/r, tipranavir boosted with ritonavir; RTV, ritonavir; FPV/r, fosamprenavir boosted with ritonavir; RAL, raltegravir; ND, not done.

RAL resistance associated mutations were identified in the ten patients: one displayed the 148 pathway, seven displayed the 155 pathway, and two displayed none of the major RAL resistance mutations, but instead the E92Q - T97A motif (patients 9 and 10, **Table 3**). No switch of resistance pathway from the 155 to the 148 or 143 pathways was observed.

**Figure 1:**
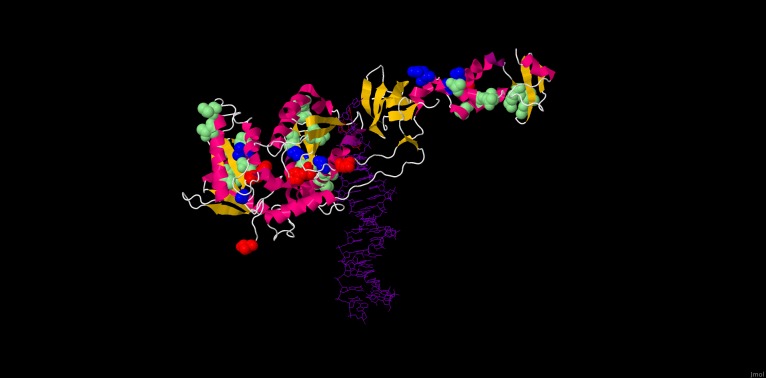
Positions selected *in vivo* under RAL pressure in an HIV-2 integrase 3D model. 3D structure of HIV-2 integrase, modelled from the Prototype Foamy Virus (PDB ID 3L2V), shown in complex with raltegravir (colored red) and DNA (colored purple). The 19 residues identified in this study as potentially associated with RAL resistance are highlighted: residues involved in major RAL resistance pathways are colored blue (92, 97, 148 and 155), and residues corresponding to minor RAL resistance mutations are colored green (44, 45, 46, 71, 84, 85, 91, 127, 151, 153, 157, 160, 163, 232 and 236). For comparison, HIV-1 active site codon positions are colored red (64, 116 and 152). The 3D structure was adapted from the pdf entry 3L2V using the JMol software (Available: http://www.jmol.org/).

Our analysis identified 19 amino acid substitutions selected in RAL-treated patients at some time point during longitudinal follow-up, and potentially emerging as secondary response to RAL pressure (**Table 3**, **Figure 1**). Fifteen amino acid substitutions occurred at non-polymorphic positions of HIV-2 wild-type: Q44H (n = 2 patients), Q45H (n = 1), K46R (n = 3), K71R (n = 1), E85K (n = 1), Q91R (n = 1), E92A/G/Q (n = 8), T97A (n = 4), K127R (n = 1), Q148R (n = 1), V151I (n = 1), N155H (n = 7), N160K (n = 2), D232N (n = 1) and K236T (n = 1). Four amino acid substitutions occurred at naturally polymorphic HIV-2 positions, but were considered in our analysis because they were previously reported as resistance associated mutations in integrase sequences from RAL-treated HIV-2 patients: I84V (n = 4), A153G (n = 3), H157N/S/R (n = 1) and S163D (n = 1). Substitutions E92A/G/Q, T97A, Q148R, A153G and N155H have been previously associated with some level of resistance to RAL on HIV-2. Substitutions E92Q, T97A, Q148R, V151I, N155H and D232N have been previously associated with INSTIs resistance in HIV-1.

The ‘148 pathway’ was observed in the virus from one patient through the presence of Q148R (patient 1, **Table 3**). No accessory mutations were detected. Mutation Q148H, the most frequently observed in HIV-1 at this position, was not present in our dataset.

The HIV-1 signature mutation N155H was observed in viruses from seven patients. Four of these patients had only one genotype available for analysis, and three patients had longitudinal genotypic data while still under RAL. Of the four patients with only one post-RAL genotype, two shared the same pattern of accessory mutations, I84V - E92A - A153G (patients 2 and 5, **Table 3**); one displayed only two of these mutations, I84V - A153G (patient 3, **Table 3**), probably due to the short exposure time to RAL; and the other displayed E92Q - T97A - S163D (patient 4, **Table 3**). Besides these accessory mutations previously described in HIV-2, viruses from these four patients also displayed amino acid substitutions in six HIV-2 non-polymorphic sites: Q44H (n = 2), Q45H (n = 1), K71R (n = 1), K127R (n = 1), D232N (n = 1) and K236T (n = 1), all previously unreported in HIV-2. The three patients with longitudinal genotypic data (patients 6, 7 and 8, **Table 3**) allowed us to investigate the evolution of HIV-2 integrase genotypic resistance profiles under continued RAL selective pressure. They all harbored the N155H mutation in association with E92Q in two cases (patients 6 and 7, **Table 3**), and with E92G in one other case (patient 8, **Table 3**). Viruses from these patients also selected the I84V substitution in one case (patient 6, **Table 3**), N160K in two cases (patients 7 and 8, **Table 3**), H157N/S/R in one case (patient 6, **Table 3**), and T97A in viruses from one of the patients who selected E92Q (patient 7, **Table 3**). In this last patient, we could see the initial selection of Q91R, its replacement by E92Q three months later, followed by the selection of T97A - N160K along with E92Q and the concurrent selection against mutation at position N155. Four substitutions at non-polymorphic HIV-2 positions were also observed in these patients' genotypes at some point during RAL selective pressure: K46R (n = 3), V151I (n = 1), L242P (n = 1) and D232N (n = 1). From all the substitutions here reported for the first time, and whose impact on RAL resistance is still unknown, K46R was clearly selected and maintained over time in these patients' viruses. The remaining substitutions were observed as single episodes in patients without longitudinal genotypic data, or were deselected over time in patients with longitudinal data (**Table 3**).

Collectively, our data revealed three distinct patterns of mutations eliciting resistance to raltegravir in HIV-2: Q148R in one patient, E92Q - T97A in two patients, and N155H - E92A/G/Q in six patients. The last patient (whose viruses selected N155H - I84V - A153G) was genotyped early in RAL failure, and we cannot exclude that that pattern of mutations will evolve to one of the previously referred patterns as the exposure time to RAL increases.

## Discussion

Studies assessing the development of resistance to raltegravir in HIV-2 infection are relatively scarce and limited in sample size. This is the first study to investigate the integrase genomic region of HIV-2 using a comprehensive number of HIV-2 clinical samples: 83 integrase sequences from 73 HIV-2 infected patients.

### HIV-2 integrase gene polymorphism

The overall rate of amino acid variation was 38.6% relatively to the HIV-2 ROD reference strain, which is a value slightly higher than the one reported by other, smaller scale, studies, where values around 27% [31], 33.1% [36], and 38% [33] have been described. This is probably related to the largest size of our sample. This variability is similar to the one reported for the HIV-1 integrase, where 37.5% [62] and 38.2% [63] of sequence variability has been observed for IN residues. The catalytic motif residues were completely conserved in our dataset, in line with what was expected in face of their essential nature for IN function [64]–[66]. The low variability tolerated at such positions agrees with what other authors have previously described [31], [33], [36] and confirms the crucial role of these amino acids in IN efficacy and viral replication.

In our RAL-naïve data we found no evidence of the primary mutations responsible for raltegravir or elvitegravir resistance in HIV-1 or HIV-2. However, two amino acid substitutions at positions implicated in HIV-1 secondary resistance to raltegravir or elvitegravir were naturally present in the sequences from these patients. Amino acid 249I had already been observed in the HIV-2 integrase by previous authors [33], [36], [67], but 206S is here reported for the first time. Furthermore, at 8 HIV-1 resistance associated positions we observed atypical amino acid substitutions (125D, 138T, 153S, 156L/P/R, 157P, 163D/G/N, 201T/V and 280S), some of which had been previously referred by others [31]. However, their full impact on HIV-2 resistance remains to be elucidated.

In our sequences from INSTI-naïve patients, we observed that some polymorphisms changed their prevalence according to prior ARV (NRTI/PI) exposure: V19M and L58V significantly increased their frequency in NRTI/PI-treated patients, while E246D and M277L significantly decreased their frequency in this group. Indeed, IN and RT are thought to interact [66]. In HIV-1, recent studies have shown that some IN polymorphisms change their prevalence according to prior NRTI exposure [63], and that there are some associations between IN mutations and RT resistance mutations in ARV-failing patients [68], [69]. This supports the hypothesis of a tight physical interaction between the viral IN and RT, and/or a potential co-evolution of some of their mutations [70].

The mechanisms of this observed difference in the prevalence of some IN polymorphisms between drug-naïve and ARV-treated patient populations, as well as the associations between specific IN and RT mutations, needs further investigation. Since the coding regions for PR, RT and IN are located in the same gene, and the three enzymes before maturation are folded together in a unique polyprotein precursor, it is conceivable that several structural interactions (also among the three different proteins) may occur at this level [63]. Furthermore, the end-product of the RT-catalysed reaction is the substrate for IN. It is also conceivable that IN mutations can appear under specific drug pressure induced by antiviral agents such as NRTIs, in order to rescue the correct binding between the RT and IN enzymes, impaired by emergence of drug resistance mutations. In addition, it is possible that IN polymorphisms are co-selected with RT resistance mutations for viral fitness compensatory rescue, or vice versa.

### HIV-2 integrase gene mutations potentially associated with RAL resistance

In the present study, of the HIV-1 RAL signature mutations already described in HIV-2, only N155H and Q148R were observed. Treatment failure of HIV-2 RAL regimens seems to be associated with the emergence of resistance mutations via two main resistance pathways, stemming from N155H and Q148R. In the two patients who did not select any of these, we only observed E92Q - T97A mutations. Since in one case we observed a de-selection of the N155H mutation under therapy in a compliant patient, we can speculate that this might be a late stage evolution of the N155H pathway. In our dataset, this pathway seems to be the preferential towards the development of RAL resistance in HIV-2 group A viruses, since N155H was the predominant mutation selected in these patients, clearly outnumbering Q148R which was observed only in one patient. This disagrees with what others have reported about the N155H pathway being observed only in patients infected with HIV-2 group B viruses [41].

In addition to these two major (Q148R and N155H) and two minor (E92Q and T97A) RAL resistance mutations, other minor mutations also associated with RAL resistance in HIV-1 were observed in HIV-2 patients failing RAL-containing regimens (V151I and D232N), along with several novel mutations previously unreported in HIV-1 or HIV-2.

In line with previous reports, the A153G substitution was always detected in the same genotype as N155H, indicating a preferential association with the 155 mutational pathway, and suggesting that this double mutation may confer higher levels of resistance to RAL than the N155H single mutant [44]–[46]. The Q91R substitution was detected in a N155H carrying genotype. To our knowledge, this is the first time that Q91R is associated with the 155 pathway in HIV-2. To date, this mutation has rarely been reported, and, when so, it was either associated with the 143 pathway, together with T97A [46], or associated with the recently described mutation I175M and conferring high levels of resistance to RAL *in vitro* [36]. Further studies are necessary to elucidate the individual impact of Q91R on HIV-2 resistance to RAL, and on the replicative capacity of the virus.

Several atypical amino acid substitutions were selected in the patients failing RAL. The majority was observed as single episodes in patients without longitudinal genotypic data, or deselected over time in patients with longitudinal follow-up. Mutation K46R, however, was selected in patients with longitudinal data and persisted, being apparently fixated in the virus genome. K46R was always selected in N155H-carrying genotypes, and may constitute a secondary mutation specific to the 155 resistance pathway in HIV-2.

For HIV-2 patients remaining on a failing RAL regimen, and for whom genotypic resistance follow-up was available, we could distinguish one of two patterns: either an accumulation of secondary RAL resistance mutations, or the replacement of pre-selected mutations by novel variants. The former was more common, being observed in two of the three patients with longitudinal follow-up (patients 6 and 8, **Table 3**). Such accumulation of mutations probably intends to overcome the fitness costs due to the acquisition of primary mutations. Patient 7, however, evidenced a different genotypic pattern of evolution, through the replacement of pre-selected variants by novel ones. In this patient, we could see the initial selection of N155H - Q91R, followed by the replacement of Q91R by E92Q and K46R, and later by the addition of T97A and N160K, with the simultaneous selection against mutation at position N155. This patient constitutes an interesting case for several reasons: it was the only patient where we could observe the replacement of pre-existing mutations by new ones; it was the first time that Q91R, a mutation never reported in HIV-1, was associated with the 155 pathway; and it was the only case where we could see the selection against major mutation N155H, albeit still under RAL pressure, and its replacement with T97A - N160K. Viruses from the remaining patients that selected the N155H mutation maintained it over time, as previously described by others [44]. The patient in question is reported as adherent to the therapy (adherence evaluated by self-reporting and pill count), making unlikely the hypothesis that this de-selection could be the result of incomplete adherence. Furthermore, the de-selection of N155H occurs after the longest exposure to raltegravir (35 months) in our dataset, suggesting that despite the high level of RAL resistance conferred by this mutation [44], the impairment it causes in viral replication may lead to its replacement by other substitutions that allow a more efficient trade-off between acquisition of resistance and fitness costs. Therefore, we can speculate that the accumulation of mutations E92Q, T97A and, eventually, K46R and N160K in this patient may be sufficient to ensure resistance to RAL without the fitness costs incurred by mutation N155H [71].

In the patients with longitudinal follow-up, no switch of resistance profile from 155 to 148 or to 143 occurred. But disparate observations have been made on this matter by different HIV-2 study groups: one group agreed with our results and reported no switch of genotypic resistance profiles in the HIV-2 IN [41], but another group reported the switch from the N155H to the Y143C resistance pathway by both population and clonal sequencing [46]. This shift of resistance pathways is well documented in HIV-1 [72], [73], but further studies are required to elucidate its occurrence in HIV-2. On the other hand, our data corroborate what has been described both in HIV-1 [35], [73] and HIV-2 [32], [42] reporting that the main pathways of RAL resistance are mutually exclusive, since two primary resistance changes were never observed in the same genotype.

Mutation Q148H, the most frequently observed in HIV-1 at this residue was not detected in our dataset. Instead, we found Q148R, which is sufficient to induce phenotypic HIV-2 RAL resistance by itself [32], [42]. Mutation G140S, usually selected with Q148R to mitigate the fitness costs incurred by this primary mutation [37], [41], [42], [74], was not observed in our study, probably due to the short exposure time of the patient to RAL (only 5 months).

E92Q was observed in our dataset either in association with N155H, or with T97A. It is known that this secondary mutation increases the level of resistance to RAL of HIV-2 mutants in a Y143C- or N155H-resistant background, playing a more important role in the HIV-2 than in the HIV-1 context [41], [42], [46]. In two other patients, viruses selected and maintained the E92Q - T97A pattern without any major mutation. One of these patients is reported to have good adherence to therapy, and no adherence data is available for the other patient. This seems to suggest that E92Q, coupled with T97A, may be sufficient to elicit resistance to RAL in HIV-2, albeit to a lesser extent than N155H or Q148R, as previously demonstrated *in vitro* in the HIV-1 IN [75]. But it may also be a late stage evolution of the N155H pathway. Also the possibility of an incomplete adherence to therapy cannot be formally excluded, since we cannot detect this bias with the self-reporting method used with these two patients.

Besides E92Q, we also found two other substitutions at residue E92: E92A, a mutation less frequently described at this residue [41], [42], was observed at two of the N155H-mutated viruses but, similarly to what happens with T97A, apparently provides no additional resistance to the virus [42]; and the atypical substitution E92G was observed once in association with the N155H pathway, as previously documented [42], [46]. Both E92A (GCA) and E92G (GGA) are not transitional amino acids from the wild-type E92 (GAA) to E92Q [CA(A/G)], as we confirmed through the analysis of their nucleotide sequences, so we can speculate that these can be true resistance mutations. However, phenotypic studies are required to confirm this hypothesis.

T97A was always selected in E92Q-carrying viruses, once also with N155H. The T97A - N155H association has been previously reported in HIV-2 [41]. A recent study suggested that T97A provides no additional resistance to viruses carrying the N155H mutation, but may improve the fitness of enzymes that would otherwise be catalytically impaired [42]. Our results suggest that T97A may have the same role in viruses carrying the E92Q mutation, although further studies are necessary to confirm this hypothesis.

## Conclusions

In conclusion, raltegravir is highly effective, but so far there is no data to allow a recommendation on when to use this compound in the context of HIV-2 infection, for a first-line or later regimen. Taken together, data retrieved from this study should help build a more robust HIV-2-specific algorithm for the genotypic interpretation of raltegravir resistance. Nevertheless, further studies are needed to elucidate the true impact of some of the mutational patterns we found in the efficacy of raltegravir.
